# The effects of spondylodiscitis on the inflammation burden in infective endocarditis

**DOI:** 10.1007/s12471-024-01908-1

**Published:** 2024-11-05

**Authors:** Esen Ulas, Mariëlle Duffels, Olivier Drexhage, Tjeerd Germans, Jiri Wagenaar, Victor Umans

**Affiliations:** 1 Department of Cardiology, Northwest Clinics, Alkmaar, The Netherlands; 2 Department of Infectious Diseases, Northwest Clinics, Alkmaar, The Netherlands

**Keywords:** Infective endocarditis, Inflammation, Spondylodiscitis

## Abstract

**Background:**

This study investigates the effects of spondylodiscitis on the inflammation burden in infective endocarditis patients.

**Methods:**

A prospective, observational study was conducted between September 2018 and October 2022 in a non-surgical teaching hospital. Patients with a definite or possible and treated as infective endocarditis were recruited from the Alkmaar Endocarditis Team meetings. Spondylodiscitis was diagnosed based on symptoms and radiological findings. The inflammation burden was defined as the area under the C‑reactive protein (CRP) curve.

**Results:**

174 consecutive patients with infective endocarditis were included (mean age 73 years, 34.5% female). Concomitant spondylodiscitis was present in 32 patients (18%), frequently associated with *Streptococcus* species (38%). At admission, the mean level of CRP was significantly higher in patients with concomitant spondylodiscitis (*p* = 0.004). The median CRP area under the curve was significantly higher in spondylodiscitis patients (4.2 × 10^6^ min.mg/l [1.2 × 10^5^ − 1.6 × 10^7^ min.mg/l] vs 2.0 × 10^6^ min.mg/l [8.7 × 10^4^ − 1.6 × 10^7^ min.mg/l], *p* < 0.001). This difference remained during the whole treatment period. At 6 months of follow-up, rates of mortality and relapse of infective endocarditis were not significantly different.

**Conclusion:**

The prevalence of spondylodiscitis in non-referred patients with infective endocarditis was 18%. Endocarditis patients with spondylodiscitis had an increased inflammation burden at and during admission. This difference in normalisation of CRP levels was particularly apparent in the final phase of antibiotic treatment but not related to infectious complications. Despite an augmented inflammation burden, spondylodiscitis was not associated with mortality, cardiac surgery or infectious relapse.

**Supplementary Information:**

The online version of this article (10.1007/s12471-024-01908-1) contains supplementary material, which is available to authorized users.

## What’s new?

- This paper describes the effect of concomitant spondylodiscitis in infective endocarditis in a non-referred consecutive series of 174 patients.
- Patients with concomitant spondylodiscitis have a larger inflammation burden with a different CRP normalisation trajectory.
- This understanding may guide physicians in preventing extra imaging techniques in such stable infective endocarditis patients with spondylodiscitis.
- Despite an augmented inflammation burden, spondylodiscitis is not associated with mortality, cardiac surgery or infectious relapse.

## Introduction

Infective endocarditis is an infection of the endocardial surface of the heart and is associated with a 1-year mortality rate of up to 37% [1–5]. Its diagnosis is based on a spectrum of clinical manifestations, including extracardiac symptoms such as secondary musculoskeletal infection [2].

Spondylodiscitis is a bacterial spine infection, involving one or more vertebral bodies and vertebral disks [6]. The pathogenesis depends on the mechanism of infection: direct inoculation following trauma or surgery, contiguous spread from an infected adjacent organ or metastatic spread from a distant site [6]. In patients with infective endocarditis, spondylodiscitis is believed to be related to haematogenous bacterial spread from the heart to the highly vascular bone marrow of the vertebral bodies [6–8].

The prevalence of spondylodiscitis in infective endocarditis is increasing due to changes in the population, i.e. age, in the number of patients with (non)cardiac protheses and due to improved sophisticated imaging techniques [9]. With ageing, the vascular anatomy of the vertebrae changes and results in more sludging of the blood flow in the metaphysis of the vertebrae [10]. This alteration predisposes haematogenous bacterial seeding and makes spondylodiscitis more common in the older patient population [6]. The current incidence of spondylodiscitis in general varies from 5 to 11 cases per 100,000 persons annually [11].

The number of implanted (non)cardiac protheses is steadily rising and these devices are prone to be infected in the case of bacteraemia or organ infection. The implants facilitate the adherence of bacteria, which may lead to biofilm formation that efficiently protects them from elimination [12]. Therefore, patients with implants have an increased risk of infective endocarditis, also late after implantation [12, 13].

However, additional evidence on the implications of concomitant spondylodiscitis is currently lacking because data are being restricted to case reports or limited studies from tertiary centres [9, 14–19]. With the current rise in the incidence of infective endocarditis and the ageing population, it is important to have insight into the consequences of this extracardiac manifestation on the clinical course of infective endocarditis. Knowing that inflammatory parameters may persist in cases of concomitant spondylodiscitis might help avoid unnecessary additional imaging.

Therefore, we have investigated the impact of spondylodiscitis on the extent and normalisation of the inflammation burden in infective endocarditis patients from a prospective registry in a non-surgical teaching hospital.

## Patients and methods

### Study design

A prospective, observational study was carried out at Northwest Clinics in the Netherlands between September 2018 and October 2022. Patients were prospectively followed after being diagnosed by the Alkmaar Endocarditis Team. Data were collected prospectively from the electronic patient records and the database at the Department of Cardiology. Northwest Clinics is a non-tertiary teaching hospital serving 450,000 inhabitants.

### Patient population

All consecutive patients with definite or possible and treated as infective endocarditis according to the Modified Duke University criteria for infective endocarditis were included [2]. Apart from transthoracic and oesophageal echocardiography, the diagnostic work-up for suspected infective endocarditis also included advanced imaging: cardiac computed tomography angiography (CCTA), ^18^F‑fluorodeoxyglucose positron emission tomography CT (^18^F‑FDG-PET/CT) or magnetic resonance imaging (MRI) according to the guidelines [2].

The diagnosis of spondylodiscitis was based on symptoms with radiological signs on CT, MRI and/or ^18^F‑FDG-PET/CT. These patients were seen weekly by an orthopaedic surgeon. Embolic events were defined as (sub)clinical wedge-shaped lesions shown on any imaging modality [14].

### Aim

The primary aim of this study was to study the effect of spondylodiscitis on the inflammation burden in patients with infective endocarditis and additionally included rates of cardiac surgery, mortality and relapse of infective endocarditis within 6 months.

### Inflammation burden

Inflammation burden was defined as the area under the C‑reactive protein (CRP) curve over time in min.mg/l. Additionally, CRP levels at admission also represented the inflammation status at the onset of treatment.

### Treatment and follow-up

All patients received an empirical and targeted 4–6 week antimicrobial treatment for infective endocarditis. Repetitive blood cultures were drawn until the first negative culture. CRP measurements were performed daily during the initial 14 days and weekly thereafter until end of treatment or transfer to surgery if needed. The duration of intravenous treatment was 6 weeks when spondylodiscitis was involved. Treatment was completed in an inpatient or outpatient setting. Ambulatory, outpatient treatment was conducted according to the Endocarditis@Home guidelines of Northwest Clinics [20]. Severe cases that required surgical intervention were referred to the tertiary hospital. Post-intervention, these patients were re-admitted to Northwest Clinics for clinical rehabilitation and afterwards monitored for 30 days with regard to mortality and relapse of infective endocarditis.

### Statistical analysis

Continuous data were described as means with standard deviations and analysed through an unpaired t‑test, log transformation or a non-parametric test, such as the Mann-Whitney U test. Categorical data were described as numbers and percentages. Chi-squared tests were used for the analysis of categorical and dichotomous variables. Outcome measures were corrected for possible confounding covariates through multivariate analyses. Cox-regression analysis was specifically performed to analyse the duration of hospitalisation. All analyses were performed using SPSS software (SPSS, Inc, Chicago, IL) and a *p*-value below 0.05 was considered to be statistically significant.

### Alkmaar endocarditis team

The diagnosis of endocarditis can be challenging and requires the expertise of different medical specialists. To prevent a delay in diagnosis and starting optimal therapy we initiated the Alkmaar Endocarditis Team weekly meetings with imaging cardiologists, a microbiologist, nuclear physician and infection internist conform the ESC guidelines [2, 14]. Cardio-surgical expertise is step-wise organised. Firstly, the surgeon visits our clinic weekly and may, on request, visit patients to ascertain operability. Complicated cases are presented in the tertiary meeting. Finally, the surgeons are on call in case of emergency needs.

## Results

### Patient population

From 15 September 2018 to 19 August 2022, 174 consecutive patients were diagnosed with infective endocarditis, 32 of whom (18%) had concomitant spondylodiscitis. The majority of these patients were male (66%) with a mean age of 73 ± 12 years (Tab. 1). Of all the patients, 25 and 29% had pre-existing cardiac devices and prosthetic valves, respectively. Affected valves included the aortic in 89 (51%) and mitral in 41 cases (24%). The prosthetic valve was affected in 44 cases (8 mechanical and 36 biological valves). A total of 27 patients (16%) had cardiac device-related infectious endocarditis.

**Table 1:** Overview of baseline characteristics of patients with infective endocarditis

	With concomitant SD (*n* = 32)	Without concomitant SD (*n* = 142)	*P*-value
**Demographic variables**			
Mean age ± std. (range)	75.5 ± 9.3 (49–89)	72.1 ± 12.1 (21–93)	0.196
Sex, female (%)	11 (34.4)	49 (34.5)	0.989
**Predisposing heart conditions**			
History of IE (%)	5 (15.6)	10 (7.0)	0.157
Pacemaker (%)	10 (31.3)	34 (23.9)	0.390
Prosthetic valve (%)	9 (28.1)	41 (28.9)	0.933
**Laboratory data at hospital arrival**			
Mean CRP level ± std. (mg/l)	173.9 ± 109.4	116.8 ± 87.9	0.004
Area under CRP curve (min.mg/l)*	4.2 × 10^6^ [1.2 × 10^5^ − 1.6 × 10^7^]	2.0 × 10^6^ [8.7 × 10^4^ − 1.1 × 10^7^]	< 0.001
Mean haemoglobin level ± std. (mmol/l)	6.7 ± 1.2	7.3 ± 1.2	0.011
Mean leukocyte level ± std. (x 10^9^/l)	12.7 ± 8.1	12.1 ± 6.8	0.657
**Echocardiographic findings**			
Cardiac involvement			
*Aortic valve (%)*	14 (43.8)	76 (53.5)	0.318
– Native (%)	6 (18.8)	36 (25.4)	
– Bioprosthetic (%)	5 (15.6)	31 (21.8)	
– Mechanical (%)	3 (9.4)	5 (3.5)	
– Double valve (%)	0	1 (0.7)	
– Including (%)	0	1 (0.7)	
*Mitral valve (%)*	10 (31.3)	31 (21.8)	0.257
– Native (%)	10 (31.3)	26 (18.3)	
– Bioprosthetic (%)	0	2 (1.4)	
– Mechanical (%)	0	0	
– Double valve (%)	0	1 (0.7)	
– Including lead (%)	0	1 (0.7)	
*CDRIE (%)*	3 (9.4)	24 (16.9)	0.419
*No signs of IE (%)*	5 (15.6)	11 (7.7)	0.179
Vegetation size ≥ 10 mm (%)	3 (9.4)	17 (12.0)	0.821
**Causative organisms in blood cultures**			
Gram positives (%)	32 (100)	125 (88.0)	0.045
– Streptococci variants	12 (37.5)	51 (35.9)	0.866
– Staphylococcus aureus	8 (25.0)	33 (23.3)	0.832
– Staphylococcus epidermidis	2 (6.3)	6 (4.2)	0.641
– Enterococcus faecalis	9 (28.1)	21 (14.8)	0.071
– Other gram-positives	1 (3.1)	15 (10.6)	0.311
Gram negatives (%)	0	7 (4.9)	0.352
Negative blood cultures (%)	0	10 (7.0)	0.211
**Presumed source of infection**			
Digestive system (%)	4 (12.5)	14 (9.9)	0.747
Skin and soft tissue (%)	4 (12.5)	23 (16.2)	0.789
Odontogenic (%)	4 (12.5)	29 (20.4)	0.302
Urinary tract (%)	4 (12.5)	16 (11.3)	0.766
Other (%)	1 (3.1)	10 (7.0)	0.692
Unknown (%)	15 (46.9)	50 (35.2)	0.218
**Sites of embolisation**			
Cerebral (%)	6 (18.8)	12 (8.5)	0.106
Coronary (%)	0	8 (5.6%)	0.354
Lungs (%)	2 (6.3)	7 (4.9)	0.671
Visceral (%)	1 (3.1)	11 (7.7)	0.698

*CDRIE* cardiac device related infectious endocarditis, *CRP* C-reactive protein, *IE* infective endocarditis, *SD* spondylodiscitis, *std.* standard deviation, * median and range

### Concomitant spondylodiscitis

Spondylodiscitis was confirmed by radiological characteristics on CT scanning (1 case), MRI (3 cases), ^18^F‑FDG-PET/CT (16 cases) and MRI and ^18^F‑FDG-PET/CT combined in 12 cases.

Spinal abscesses, vertebral collapses and neurological impairment occurred in 9 (28%), 2 (6%) and 1 case(s) (3%), respectively. One patient had vertebral surgery for vertebral collapse and neurological impairment with abscess drainage (*n* = 1).

Demographic variables and predisposing heart conditions were not significantly different in patients with or without spondylodiscitis. The valves affected were comparable between the two groups and cases with large-sized vegetation were not significantly different.

The prevalence of spondylodiscitis was comparable for patients with and without systematic embolisation (9/38 (19%) vs 23/104 (1%), *p* = 0.875). A total of 100 cases (58%) eventually continued the antibiotic treatment in an ambulatory setting, the majority of which were not diagnosed with concomitant spondylodiscitis (13 vs 87 patients, *p* = 0.033).

### Inflammation burden

**Fig. 1:**
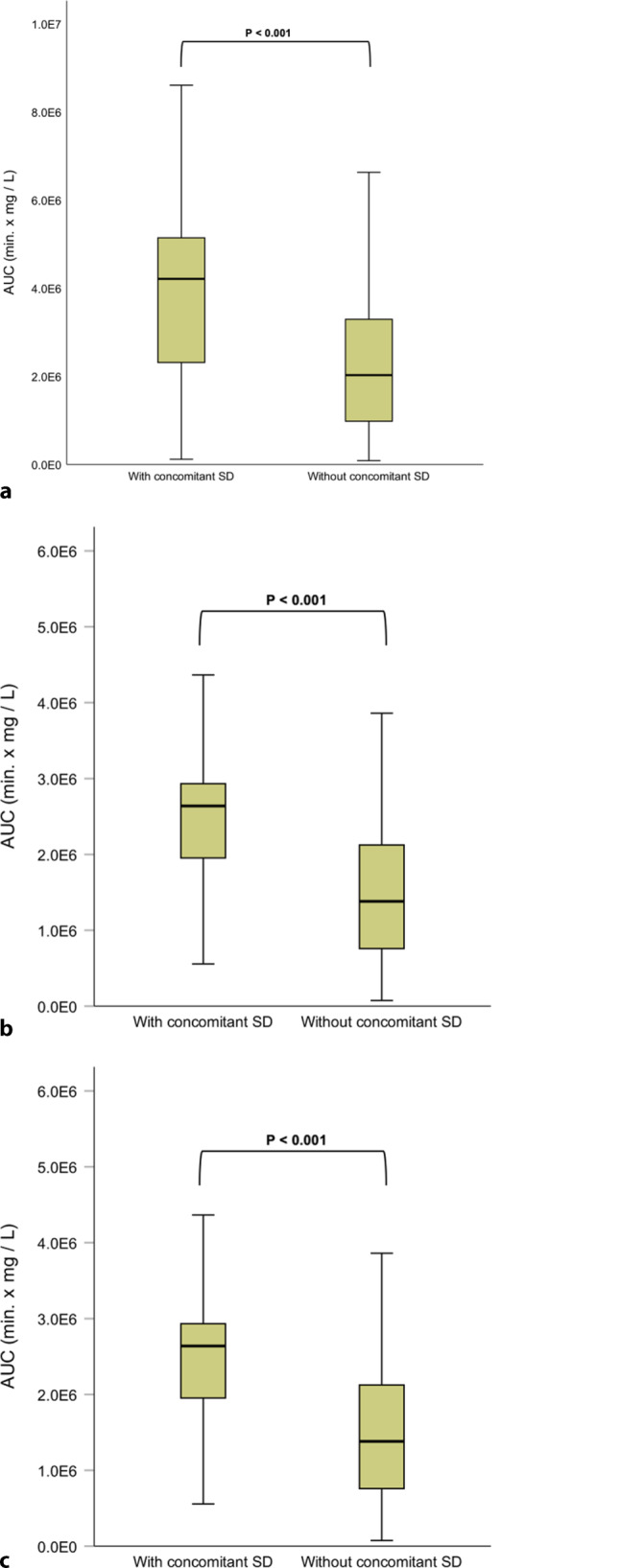
Boxplots for the median area under the curve (AUC) of CRP levels during hospitalisation. **a** Total duration of hospitalisation; **b** First 14 days of hospitalisation; **c** After 14 days of hospitalisation. *SD* spondylodiscitis

On arrival to hospital, patients with infective endocarditis and concomitant spondylodiscitis had a significantly higher CRP level compared with patients without spondylodiscitis (173.9 ± 109.4 vs 116.8 ± 87.9, *p* = 0.004). Differences in leucocyte levels at admission were not statistically significant for infective endocarditis patients with and without concomitant spondylodiscitis (*p* = 0.657). Per protocol, a median of 16 vs 12 CRP measurements (spondylodiscitis vs none) were performed during the course of treatment with a median time interval of 2880 min (2 days) in both groups. The median CRP area under the curve was significantly higher in spondylodiscitis patients (4.2 × 10^6^ min.mg/l [1.2 × 10^5^ − 1.6 × 10^7^ min.mg/l] vs 2.0 × 10^6^ min.mg/l [8.7 × 10^4^ − 1.6 × 10^7^ min.mg/l], *p* < 0.001). This difference remained in the first 2 weeks but, importantly, also in the period after 14 days (Fig. 1).

All cases with concomitant spondylodiscitis were associated with gram-positive bacteria (Tab. 1). There were relatively more cases of concomitant spondylodiscitis associated with bacteraemia by *Enterococcus faecalis* (28% vs 14%, *p* = 0.071).

### Outcome measures

Mortality, cardiac surgery and lead extraction were not significantly different between the two groups and spondylodiscitis was not associated with more systemic embolisation (Tab. 2).

**Table 2:** Short- and long-term outcome measures in patients with infective endocarditis

	With concomitant SD (*n* = 32)	Without concomitant SD (*n* = 142)	Effect size (95% CI)	*P*-value
*Clinical outcome measures*				
Hospitalisation (days ± std.)	47.0 ± 18.5	43.4 ± 17.4	HR 1.423 (0.958–2.112)	0.080
Cardiac surgery (no. %)	3 (9.4)	17 (12.0)	OR 0.687 (0.174–2.707)	0.592
Lead extractions (no. %)	1 (3.1)	17 (12.0)	OR 0.184 (0.022–1.543)	0.119
Clinical mortality (no. %)	5 (15.6)	18 (12.7)	OR 1.535 (0.441–5.349)	0.501
*Follow-up at 6 months*				
Mortality (no. %)	2 (7.4)	14 (11.3)	OR 0.965 (0.241–3.873)	0.960
Relapse of IE (no. %)	6 (22.2)	12 (9.68)	OR 2.873 (0.873–9.461)	0.083

*CI* confidence interval, *HR* hazard ratio, *IE* infective endocarditis, *no*. number of patients, *OR* odds ratio, *SD* spondylodiscitis, *std*. standard deviation

Duration of hospitalisation for all the patients varied from 1 to 95 days with a median of 24 days (IQR 25). For nine patients the duration was shorter than 14 days due to clinical death as a result of rapid progression of infective endocarditis. Patients with concomitant spondylodiscitis were hospitalised longer although this was not statistically significant (Tab. 2).

Mortality and relapse within 6 months after discharge were not significantly different between the groups. The initial cases with relapse of infective endocarditis were most frequently associated with *Streptococcus* species as causative microorganisms (62%).

## Discussion

In the last decades, knowledge on the clinical course, diagnosis and treatment of infective endocarditis has substantially increased. Despite these developments, data on the association between infective endocarditis and spondylodiscitis in non-referred patients are still lacking. The presence of spondylodiscitis (18%) in this all-comer registry shows an increased inflammation burden at and during admission. The observed difference in normalisation of CRP levels is particularly apparent in the final phase of antibiotic treatment but not related to infectious complications. Knowledge of this delayed normalisation of the inflammation burden is important in considering the need for additional imaging in such patients.

### *Clinical outcome*

In the present series, spondylodiscitis occurred in 18% of the patients and was primarily accompanied by back pain at presentation. Its presence was not related to the observed ratio of distal organ embolisation. Earlier we reported a distant embolisation rate in non-referred patients of 24% [14].

Unfortunately, the ESC-ENDO Registry [21, 22] did not register spondylodiscitis; however, their overall PET-CT experience showed a spinal uptake in 21.8% of patients who underwent a PET scan. It is of note that the use of PET-CT is very variable in the registry (mean 16.6% range 2.1–33.9%) and in the literature, which may be an important caveat in determining the prevalence of spondylodiscitis.

Urgent surgery for endocarditis during hospitalisation was infrequent and not associated with spondylodiscitis. When needed, surgery in spondylodiscitis patients was performed later than in non-spondylodiscitis patients (36 ± 9 days vs 15 ± 12 days; *p* = 0.007). Mortality and the relapse rate of infective endocarditis at 6 months was not different when spondylodiscitis was present.

### C-reactive protein and infective endocarditis

CRP is an acute-phase reactant protein that is produced by the liver in response to inflammation. It is commonly used as a marker of inflammation and can help monitor disease activity, assess response to treatment and predict prognosis in various inflammatory conditions. Our admission CRP and leucocytes levels were comparable if not higher than those found in the ESC-ENDO Registry [21, 22] which does not report follow-up CRP measurements. Heiro et al. [23] report a significant trend between the level of CRP at admission and both short-term and 1‑year outcome. In the trend analysis of association, it was found that a CRP increment of 50 mg/l at admission was associated with a 1.33-fold hazard for in-hospital death (HR 1.33, 95% CI 1.06 to 1.68; *p* = 0.015) Among their surgically treated patients, the hazard for in-hospital death for the patients with CRP values ≥ 100 mg/l at admission was 6.85-fold as compared with those with CRP values < 100 mg/l at admission (HR 6.85, 95% CI 1.51 to 30.95; *p* = 0.013).

Serial CRP measurements are valuable in infective endocarditis as they allow us to monitor disease activity over time. Changes in CRP levels and leucocyte levels can reflect fluctuations in the inflammatory process, providing insights into the effectiveness of treatment or disease progression [24]. Guideline-driven antibiotic therapy indeed shows a gradual normalisation of these levels in uncomplicated cases. However, spondylodiscitis influences the CRP levels both at admission and during the treatment period with slower normalisation. The second consequence of spondylodiscitis is the duration of intravenous treatment: always 6 weeks irrespective of the micro-organism. The clinical follow-up thereafter is similar for patients with and without spondylodiscitis and finally, we did not observe more relapses in the two groups.

### Systemic inflammation burden

In contrast to previous retrospective studies with smaller sample sizes, the current study reports a significantly higher admission level of CRP and lower level of haemoglobin in patients with concomitant spondylodiscitis when compared with patients with infective endocarditis alone [14, 26]. Additionally, the inflammation burden (denoted as CRP area under the curve) during the course of the disease is larger in spondylodiscitis patients. This indicates that patients with concomitant spondylodiscitis are going through a more severe systemic infection, also given their higher leucocyte levels.

Tracking the inflammation burden in infective endocarditis can provide valuable information about disease activity, treatment effect and prognosis. Elevated and persistent CRP levels with or without fever may reflect ongoing disease activity, and renewed CRP elevation in the later phase may reflect new inflammatory activity, more likely other than endocarditis. Our study demonstrates the value of monitoring the CRP area under the curve when spondylodiscitis itself shows a prolonged period of elevated CRP levels without endocarditis complications. Recognition of this different pattern of CRP normalisation in both the initial and later clinical phase may guide physicians in their awareness that when the clinical condition remains stable, it is not necessary to perform immediate new cardiac imaging for suspicion of infectious valve complications.

Fortunately, the increased inflammation burden does not interfere with the duration of hospital stay, the rates of cardiac surgery and mortality. It seems that once targeted therapy is started, the spondylodiscitis can be treated equally effectively, albeit slower.

Interestingly, we report a trend towards *Enterococcus*-mediated spondylodiscitis in our unselected population. *Enterococci* are the third most common species to cause infective endocarditis [24, 25] and have a large impact on patients due to the development of a protective bacterial film [11, 12] leading to increased inflammation burden in endocarditis patients. They are more frequently found in elderly patients and particularly in the hospital environment mostly due to urinary and bowel interventions. Finally, they have intrinsic resistance to some antibiotics [26, 27].

### Limitations

There are several limitations that need to be taken into account. The main limitation of this study is its single-centre nature, which resulted in a relatively small group of patients with infective endocarditis. Also, 16 patients without valve abnormalities are reported because they were clinically treated as having endocarditis (Table S1, S2 of the Electronic Supplementary Material). Secondly, it is important to mention that the duration of hospitalisation for patients receiving ambulatory treatment was calculated from the day of admission until the end of the outpatient antibiotic treatment. Thirdly, not all patients underwent CT imaging, which could lead to spondylodiscitis being undetected.

Lastly, the inflammation burden expressed as the area under the CRP curve was calculated from time intervals which were determined in daily practice.

## Conclusion

In conclusion, the prevalence of spondylodiscitis in non-referred endocarditis patients was 18%. Concomitant intervertebral infection leads to higher admission levels of CRP and a larger inflammation burden with a larger area under the CRP curve. Recognition of a different CRP normalisation curve, which is not related to valve complications, is fundamental in preventing unnecessary use of additional imaging modalities. The larger inflammation burden in concomitant spondylodiscitis is not associated with a longer hospital stay, higher rates of cardiac surgery, infectious relapse or mortality.

## Supplementary Information

**Table S1. **Overview of Complications of Patients with Infective Endocarditis.

**Table S2. **Overview of Baseline Characteristics of Patients without Echocardiographic Signs for IE.
