# Supplementary material for: The effects of spondylodiscitis on the inflammation burden in infective endocarditis
Source: Neth Heart J. 2024 Nov 5;32(12):455–61. doi: 10.1007/s12471-024-01908-1 (PMC11584835; doi:10.1007/s12471-024-01908-1)
Supplement: Supplementary file 1 — Table S1. Overview of Complications of Patients with Infective Endocarditis. [file 12471_2024_1908_MOESM1_ESM.docx]

**Table S1.** *Overview of Complications of Patients with Infective Endocarditis.*

|  | **With concomitant SD**  **(N = 32)** | **Without concomitant SD**  **(N = 142)** |
| --- | --- | --- |
| **Complications** | | |
| Distant embolization (%) | 9 (28.1) | 38 (26.8) |
| Progression size of vegetation (%) | 1 (3.1) | 2 (1.4) |
| Presence of abscesses, fistulas and/or aneurysms (%) | 1 (3.1) | 7 (4.9) |
| Persistent infection (%) | 1 (3.1) | 2 (1.4) |
| *Abbreviations: SD, spondylodiscitis.* | | |
