# Supplementary material for: The effects of spondylodiscitis on the inflammation burden in infective endocarditis
Source: Neth Heart J. 2024 Nov 5;32(12):455–61. doi: 10.1007/s12471-024-01908-1 (PMC11584835; doi:10.1007/s12471-024-01908-1)
Supplement: Supplementary file 2 — Table S2. Overview of Baseline Characteristics of Patients without Echocardiographic Signs for IE. [file 12471_2024_1908_MOESM2_ESM.docx]

**Table S2.** *Overview of Baseline Characteristics of Patients without Echocardiographic Signs for IE.*

|  | **Patients without echocardiographic signs for IE (N= 16)** | |
| --- | --- | --- |
| **Demographic variables** | | |
| Mean age ± std. (range) | 73.8 ± 8.9 (45-84) | |
| Sex, female (%) | 7 (43.8) | |
| **Predisposing heart conditions** | | |
| History of IE (%) | 1 (6.3) | |
| Pacemaker (%) | 6 (37.5) | |
| Prosthetic valve (%) | 6 (37.5) | |
| **Causative organisms in blood cultures** | | |
| Gram positives (%) | 16 (100) | |
| *Streptococci variants* | 6 (37.5) | |
| *Staphylococcus aureus* | 6 (37.5) | |
| *Staphylococcus epidermidis* | 0 | |
| *Enterococcus faecalis* | 3 (18.8) | |
| *Other gram-positives* | 1 (6.3) | |
| Gram negatives (%) | 0 | |
| Negative blood cultures (%) | 0 | |
| **Sites of embolization** | | |
| Cerebral (%) | 1 (6.3) | |
| Coronary (%) | 1 (6.3) | |
| Lungs (%) | 0 | |
| Visceral (%) | 1 (6.3) | |
| *Abbreviations: IE, infective endocarditis; std., standard deviation.* | |  |
